# A case of primary pulmonary synovial sarcoma

**DOI:** 10.1016/j.rmcr.2025.102236

**Published:** 2025-05-23

**Authors:** Keigo Ozono, Keita Sakanashi, Naoya Iwamoto, Yoshiyuki Nakanishi, Yoshinao Oda, Masafumi Nakamura

**Affiliations:** a Department of Surgery and Oncology, Kyushu University, Fukuoka, Japan; b Anatomic Pathology, Kyushu University, Fukuoka, Japan

**Keywords:** Synovial sarcoma, Lung, PPSS, Pulmonary malignancy

## Abstract

**Background:**

Primary pulmonary synovial sarcoma (PPSS) is an extremely rare pulmonary malignancy and often poses diagnostic challenges, especially in differentiating it from benign tumors.

**Case presentation:**

A 46-year-old woman presented with a slowly enlarging, well-defined nodule in the right lower lobe of the lung. Positron emission tomography showed low FDG uptake (SUVmax 1.52), raising suspicion for a benign lesion. However, histopathologic examination following wedge resection revealed primary pulmonary synovial sarcoma. Subsequently, a right lower lobectomy was performed. The patient has remained recurrence-free for over one year.

**Conclusions:**

Surgical resection with clear margins is essential in the management of PPSS. This case highlights the difficulty in distinguishing PPSS from benign lung lesions based on imaging alone, underscoring the importance of pathological confirmation. Accumulation of more reported cases is needed to develop standardized treatment strategies.

## 1 Background

Synovial sarcoma commonly occurs around the large joints of the extremities in young adults, and it is considered a carcinosarcoma of unknown tissue origin that develops in soft tissues. Primary pulmonary sarcoma accounts for only 0.1–2 % of malignant lung tumors, and among all of the primary pulmonary neoplasms, primary pulmonary synovial sarcoma (PPSS) accounts for approx. 0.1 % [1]. The 5-year survival rate of patients with PPSS has been only 46 %, indicating a poor prognosis [2]. We present the case of an adult woman with a PPSS that was resected. The tumor was located in the lower lobe of the right lung, and we describe the histopathological findings.

## 2 Case

**Fig. 1:**
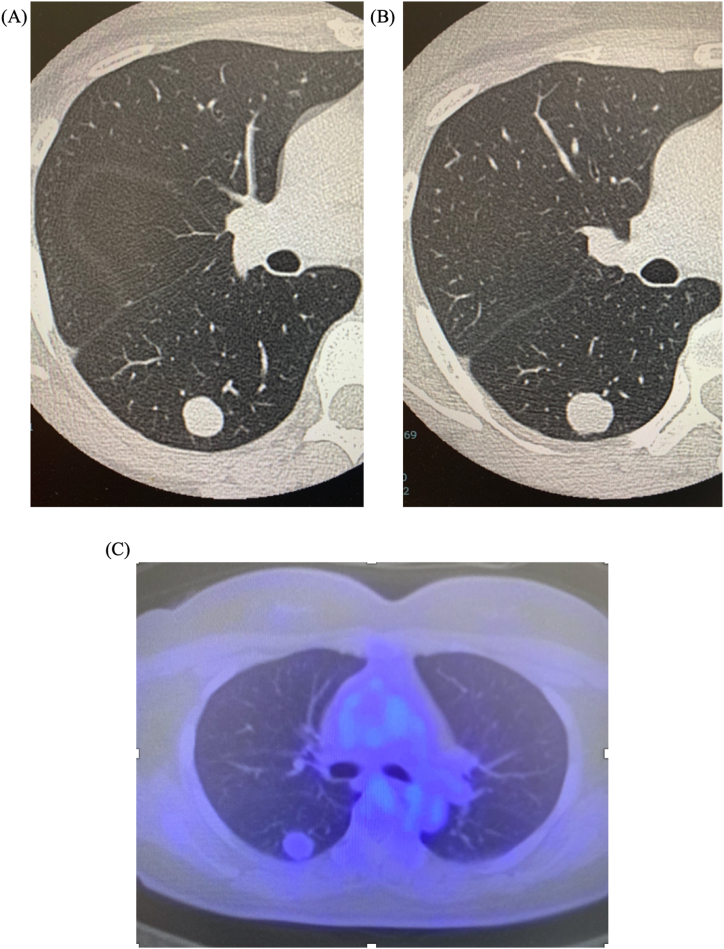
The preoperative image findings of the patient, a 46-year-old woman. **(A)** Computed tomography revealed a well demarcated 14-mm solid lung tumor in the right lower lobe (*arrowheads*). **(B)** A follow-up CT image ∼12 months later showed a slight increase in the tumor's size to 16 mm. **(C)** PET-CT indicated FDG accumulation with an SUV of 1.52 at the tumor, with no significant accumulation at any other site (*arrowheads*).

A 46-year-old Japanese woman was referred to our hospital's outpatient clinic due to an abnormality observed on chest computed tomography (CT). One year prior to that examination, an abnormal shadow was noted on a chest X-ray in a medical checkup, and a CT scan at the previous hospital revealed a well-demarcated 14-mm tumor at the right lung's lower lobe. Based on its morphology, a benign tumor was diagnosed, and a follow-up CT scan one year later showed that the tumor's size had increased to 16 mm (Fig. 1A and B).

At the patient's presentation in our hospital, the physical examination revealed no special findings, and her performance status (PS) was 0. Positron emission tomography (PET)-CT showed fluorodeoxyglucose (FDG) accumulation with a standard uptake value (SUV) of 1.52 at the tumor site, with no other areas of significant accumulation (Fig. 1C). Brain contrast magnetic resonance imaging (MRI) revealed no metastasis. Blood chemistry results showed no elevated level of the tumor markers carcinoembryonic antigen (CEA), cytokeratin 19 fragment (CYFRA), neuron-specific enolase (NSE), squamous cell carcinoma related antigen (SCC), and pro-gastrin releasing peptide (pro-GRP).

**Fig. 2:**
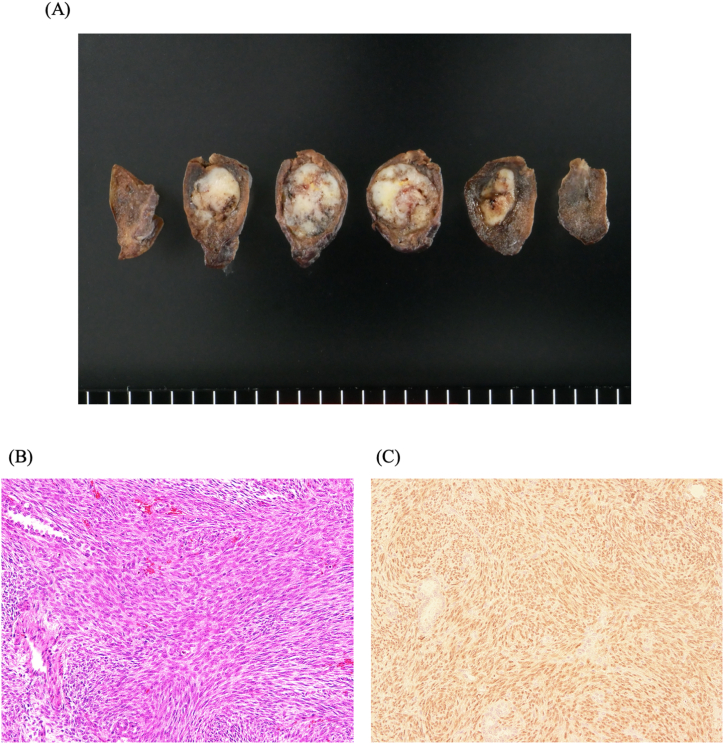
Macroscopic and microscopical findings. **(A)** Macroscopically, the cut surface revealed a whiteish, solid tumor. **(B)** Microscopically, H&E staining showed spindle-shaped tumor cells with hyperchromatic nuclei arranged in a fascicular pattern, along with entrapped alveolar epithelial cells ( × 200). **(C)** Immunohistochemically, the tumor cells were positive for SS18-SSX ( × 200).

Based on the morphology and the degree of FDG accumulation, we considered the possibility of a benign lesion to be high and thus performed video-assisted thoracic surgery (VATS) for partial resection of the right lower lobe. The postoperative course was uneventful, and the thoracic drain was removed on postoperative day (POD)1. The patient was discharged on POD7 without serious complication. Macroscopically, the cut surface of the mass showed a whiteish, well-demarcated solid tumor (Fig. 2A). Microscopically, hematoxylin and eosin staining showed a 15 × 12 × 11-mm tumor composed of spindle-shaped tumor cells with hyperchromatic nuclei arranged in a fascicular pattern with entrapped alveolar epithelial cells (Fig. 2B).

Immunohistochemically, the tumor cells were positive for SS18-SSX (Fig. 2C), AE1/AE3, EMA, D2-40, calretinin, and p16 but negative for alpha-SMA, desmin, S-100 protein, SOX10, CD34, WT1, and MDM2 (Table 1). Alveolar epithelial cells were positive for TTF-1. We made the diagnosis of PPSS based on these findings, and after a thorough discussion the patient's informed consent for a remnant lobectomy was obtained. Two weeks after the initial surgery, we performed a VATS right lower lobectomy with ND2a-2 lymph node dissection.

**Table 1:** Immunohistochemical staining results.

Positive	Negative
SS18-SSX	Desmin
AE1/AE3	α-SMA
EMA	S-100
D2-40	SOX10
Calretinin	CD34
P16	WT1
	MDM2

The postoperative course was relatively smooth. The drain was removed on POD2. On POD5, the patient developed a mild case of pneumonia, which was managed with oral antibiotics and close monitoring. She also experienced a mild cough, for which antitussive medication was prescribed. The patient was discharged on POD14.

The final pathological results revealed no residual lesions in the remaining lower lobe, and no lymph node metastases. The patient has been recurrence-free for >1 year and is currently undergoing regular outpatient follow-up.

## 3 Discussion

Primary pulmonary sarcoma accounts for only 0.1 %–2 % of malignant lung tumors. Among them, the frequency of PPSS accounts for approx. 0.1 % [1]. The typical age of PPSS onset ranges from young to middle adulthood, with no significant gender differences observed [3]. Symptoms are rare [4]. It is also reported that PPSS frequently metastasizes to hilar or mediastinal lymph nodes [5], and that the average survival of the patients was 16.1 months (median 12 months) [6].

Imaging findings often reveal calcification within the internal structures of a PPSS, particularly on CT scans [7]. On MRI, three distinct areas can be identified on T2-weighted images: high signal intensity indicating necrosis or fluid, a relatively high signal indicating blood, and a low signal indicating calcification. This creates a characteristic observation known as triple signal intensity. A fluid-fluid level may be observed [4]. However, in cases without calcification (as in the present patient's case), differentiating the tumor from benign lesions can be challenging, as benign tumors often exhibit a well-defined tumor image. With regard to PET-CT, the FDG uptake in synovial sarcoma often results in SUVmax values > 10, reflecting the high metabolic activity that is characteristic of highly malignant tumors. This pattern of FDG accumulation is commonly associated with aggressive tumor behavior and is used to assess malignancy and monitor treatment response [8]. However, in our patient's case, the FDG accumulation was poor (SUVmax 1.52). This led to the conclusion that the nodule was benign; however, it must be noted that there are cases of synovial sarcoma without calcification and with poor FDG uptake.

The SS18-SSX immunohistochemistry was positive in our patient's case, suggesting the presence of the SS18-SSX fusion gene, leading to the diagnosis of synovial sarcoma. This fusion gene is used as a specific marker for diagnosing synovial sarcoma. The majority of synovial sarcomas (>90 %) carry the t(X; 18) chromosomal translocation, which results in the fusion of the SS18 gene (SYT) and the SSX gene. The identification of this fusion gene is crucial for distinguishing synovial sarcoma from other soft tissue sarcomas or similar tumors [9].

Regarding treatment, chemotherapy combining ifosfamide and doxorubicin or local radiation therapy is administered in cases with residual tumors or unresectable cases [10]. However, the number of reported PPSS cases is extremely limited, making it difficult to establish a definitive treatment protocol. The primary approach to the treatment of PPSS remains the complete resection of the tumor [11]. Nevertheless, reports indicate a local recurrence rate as high as 70 % [12].

Even with surgical excision, it is advisable to choose a surgical technique that ensures a sufficient margin from the tumor to the resection margin, rather than a local excision. In our patient's case, a partial resection was performed, and the postoperative histological examination revealed findings of synovial sarcoma, although the resection margins were negative. Based on the reports of a high recurrence rate and lymph node metastasis associated with PPSS, we decided to proceed with a lobectomy and ND2a-2 lymph node dissection. Adjuvant chemotherapy was not desired by the patient, and we are thus currently following her with outpatient monitoring. The patient has been recurrence-free for >1 year.

## 4 Conclusion

The preoperative diagnosis of the rare pulmonary malignancy PPSS is difficult, especially in cases without calcification or significant FDG uptake. Surgical resection remains the critical treatment, with a focus on achieving clear margins to reduce the risk of recurrence. An accumulation of more PPSS cases is necessary before a standardized treatment protocol can be established.

## CRediT authorship contribution statement

**Keigo Ozono:** Writing – original draft, Writing – review & editing. **Keita Sakanashi:** Formal analysis. **Naoya Iwamoto:** Data curation. **Yoshiyuki Nakanishi:** Conceptualization. **Yoshinao Oda:** Supervision. **Masafumi Nakamura:** Supervision.

## Ethics approval and consent to participate

Ethics approval was not required for this case report.

## Consent for publication

Written informed consent for publication of the case details and accompanying images was obtained from the patient.

## Availability of data and materials

All data generated or analyzed during this study are included in this published article.

## Funding

This research did not receive any specific grant from funding agencies in the public, commercial, or not-for-profit sectors.

## Declaration of competing interest

The authors declare that they have no known competing financial interests or personal relationships that could have appeared to influence the work reported in this paper.

## Abbreviations

CT: computed tomography
FDG: fluorodeoxyglucose
H&E: hematoxylin and eosin
MRI: magnetic resonance imaging
PET-CT: positron emission tomography-CT
POD: postoperative day
PPSS: primary pulmonary synovial sarcoma
SUV: standard uptake value
VATS: video-assisted thoracoscopic surgery
